# NFX1, Its Isoforms and Roles in Biology, Disease and Cancer

**DOI:** 10.3390/biology10040279

**Published:** 2021-03-30

**Authors:** Sreenivasulu Chintala, Rachel A. Katzenellenbogen

**Affiliations:** Department of Pediatrics, Indiana University School of Medicine, Indianapolis, IN 46202, USA; srchinta@iu.edu

**Keywords:** human papillomavirus, human papillomavirus-associated cancers, NFX1, NFX1-123

## Abstract

**Simple Summary:**

The *NFX1* gene, and its gene products, were identified over 30 years ago. Since then, the literature on *NFX1* homologs and *NFX1* itself has grown. In this review, we summarize the studies to-date on the *NFX1* gene and its proteins across species and in humans, describing their role in gene regulation, embryonic development, cellular growth and differentiation, exogenous stress tolerance and metabolism, and an organism’s immune response. We also highlight the roles NFX1 has in human disease and in cancer, with a strong focus on its collaborative role with high-risk human papillomavirus infections that cause cervical and head and neck cancers. We believe this is the first comprehensive review of NFX1 and its functional significance in organisms ranging from yeast to human.

**Abstract:**

In 1989, two NFX1 protein products were identified as nuclear proteins with the ability to bind to X-box *cis*-elements. Since that publication, the *NFX1* gene and its homologs have been identified, from yeast to humans. This review article summarizes what is known about the *NFX1* gene across species. We describe the gene and protein motifs of *NFX1* homologs and their functions in cellular biology, then turn to *NFX1* in human biology and disease development. In that, we focus on more recent literature about *NFX1* and its two splice variants protein products (NFX1-91 and NFX1-123) that are expressed in epithelial cells. We describe new evidence of conserved protein motifs, direct and indirect gene expression regulation, and critical protein-protein interactions. Finally, we stress the emerging roles of these NFX1 splice variants in high-risk human papillomavirus-associated cancers, and the increased expression of the longer splice variant, NFX1-123, found in these cancers.

## 1. Introduction

More than three decades ago, Hume and Lee from the Memorial Sloan-Kettering Institute of New York demonstrated the binding of two nuclear protein complexes, NFX1.1 and NFX1.2, to a Class II X box consensus element [1]. In doing so, these proteins were identified and found to be repressors of HLA class II family genes [1]. Five years later, Song et al. published a complementary study that identified a novel cysteine-rich transcription factor, NF-X1. This transcription factor was a 1104 amino acid polypeptide that bound a conserved X-box motif of MHC class II genes and transcriptionally repressed gene expression [2]. This repression was shown to play a significant role in the regulation of inflammatory responses, limiting the period in which MHC class II molecules could be induced by IFN-gamma [2]. Since these two publications, foundational studies of *NFX1* and its homologs have been conducted, and they add detail to our understanding of *NFX1*, its conserved domains, and its conserved functions.

In this review, we will summarize the data to-date on: the biologic role of *NFX1* and its homologs across different species and in humans; the role *NFX1* and its homologs has in disease; and the role *NFX1* has in cancers.

## 2. NFX1 Homologs and Biologic Functions Across Species

The official name of the gene *NFX1* in humans (Gene ID 4799 at NCBI) is Nuclear Transcription Factor, X-box binding 1. It is also known as NF-X1, NFX-1, NF.X1, NFX2, Tex42, and TEG-42 in the literature. For clarity, we will use the italicized term *NFX1* for the human gene, and we will use NFX1 or isoform-specific names when discussing the human gene products. Regardless of this nomenclature, *NFX1* has homologs across species. We will focus on studies of eukaryote *NFX1* homolog lineages and findings regarding their congruent domains and typical biologic functions in each of these organisms. 

### 2.1. Stc: Drosophila Homolog Studies

In 1996 and 1998, two studies were published on the *shuttle craft* gene (*stc*) in fruit flies. *Shuttle craft* is the *NFX1* gene homolog in *Drosophila melanogaster*, and through mutational studies of *stc*, the functional importance of *NFX1* was expanded, from immune regulation noted in human cells in 1989 and 1994, to include embryogenesis [3,4]. *Stc* was found to be required for the embryonic central nervous system to develop; when *stc* was deleted, it was embryonic lethal [3,4]. However, in addition to these findings, *stc* appears to play a role in the lifespan of adult fruit flies [5,6]. Therefore, in Drosophila, the *NFX1* homolog *stc* is vital both at the start of development and in the extension of longevity during adulthood.

### 2.2. m-Nfx.1: Mouse Homolog Studies

The murine homolog of *NFX1*, *m-Nfx.1*, has been cloned, and it has significant homology to both the human *NFX1* gene as well as the Drosophila *stc* gene. Like *NFX1*, the mouse homolog contains a cysteine-rich DNA-binding domain. This shared domain, between the human and mouse genes, links the amino acid sequences to their analogous functions in cellular biology—the binding of DNA as transcription factors [7]. 

### 2.3. FAP1: Saccharomyces cerevisiae Homolog Studies

Fatty acid protein 1 (*FAP1*), the homolog of *NFX1* in *S. cerevisiae*, competes with rapamycin for binding to FKBP12, the receptor of rapamycin that confers resistance to rapamycin [8]. Although the ligand/receptor function of FAP1 and FKBP12 do not directly align with the functions of *NFX1*, NFX1 can bind to other drugs in human cells (see Section 3). Additionally, FAP1 expression was found to be inversely proportionate to the availability of nitrogen in the culture medium of S. cerevisiae [9]. Other homologs of *NFX1* (see below) also have effects on an organism’s growth regulation, either in the context of exogenous stress or with nutrients included in the diet. 

### 2.4. AtNFXL1, AtNFXL2, and NF-X1: Plant Homolog Studies

Plant models have documented the functional importance of *NFX1* homologs in growth, and specifically growth during physiologic stress. Two *NFX1* homologs, *AtNFXL1* and *AtNFXL2*, were identified in the Arabidopsis genome and found to be associated with salt and drought stress responses [10]. In Arabidopsis, the *NFX1* homolog *AtNFXL1* was also involved in acclimation to high temperatures [11], was a signaling component of the type A trichothecene-dependent response [12], and more broadly the negative regulation of phytotoxin-induced defense response [13]. All of these findings point to *AtNFXL1′s* importance in organismal survival. De novo transcriptome assembly and co-expression network analysis in *Cynanchum thesioides* revealed the *NFX1* homolog, *NF-X1*, was one of the drought stress resistance transcription factors in a xerophytic plant model [14]; again, this emphasizes the role *NFX1* homologs play in response to physiologic and exogenous stresses. Additional detailed reviews of the structure and putative function of NFX1-like proteins in plants have been published [15].

### 2.5. NFX1: Large Animal Model Studies

In complement to the plant studies and their documentation of *AtNFXL1* in response to stress, studies in large animals also demonstrated how nutritional supplements in feed affected the expression and function of NFX1. In a large animal model, lambs had a downregulation of *NFX1*, along with other genes involved in immune, inflammation, and stress pathways when provided feed mixed with the anti-inflammatory compounds cinnamon bark, dill seed, essential oils, and eucalyptus leaves. With these nutritional supplements added to feed and the associated reduction in NFX1 and other immune pathway genes, these animals had improved health [16]. 

In pigs and cattle, *NFX1* affected both cellular growth and whole-organism growth based on gene expression and pathway analyses. In pigs, *NFX1* was identified as a molecular predictor of feed efficiency in growing animals, and *NFX1* clustered with other genes included in inflammatory response gene ontology (GO) biological processes [17]. In cattle, NFX1 appears to have a role in myocyte growth. Transcriptome analyses of muscle samples of *Bos indicus* revealed *NFX1* as one of several transcription factors differentially expressed in the muscle of post-pubertal cattle [18]. Furthermore, the *NFX1* gene was identified in genomic SNP variance regions in the ribeye area of Nellore cattle, and this variance was associated with the amount of meat in the carcass and backfat regions, and with the protection of the cattle carcass meat after slaughter during cooling [19]. As a collective, these studies in large animals (lambs, pigs, and cattle) reflect a role for NFX1 in growth and metabolism. These functional findings across *NFX1* homologs—the response to inflammation, the support of development, growth, and longevity, and the protection from exogenous stress—indicate conserved biologic actions. Additionally, conserved protein motifs point to overlapping mechanisms of action by *NFX1* homologs that drive these biologic actions. These commonalities hold true in studies of the human *NFX1* gene and its gene products as well.

## 3. NFX1 and NFXL1 Biologic Functions in Humans

As noted above, the human *NFX1* gene was identified first in 1989 as a transcription factor that can bind to X-box motifs in DNA [1]. NFX1 has a cysteine-rich central domain that is the presumed DNA binding region, but it also has a PHD/RING domain at the start of its central domain. PHD/RING domains have E3 ubiquitin ligase functions, and ten years after *NFX1* was first identified, it was found, along with other RING finger-containing proteins like AO7, BRCA1, and Siah-1, to bind to ubiquitin-conjugating enzymes (E2s) [20]. This strongly suggested that *NFX1* may act as an E3 ubiquitin ligase in cells to regulate protein function and stability. This study then added post-translational modification as a function of *NFX1* in addition to transcriptional regulation via binding to promoter *cis*-elements.

In addition to FAP1 binding to the receptor for rapamycin, NFX1 has been reported as one of the paclitaxel (Taxol) anticancer drug-binding proteins. This was determined through screening of paclitaxel-binding molecules from a random peptide library using paclitaxel-photoimmobilized TentaGel resin with a partial recombinant protein of NFX1 in pull-down assays and Surface Plasmon Resonance analyses [21].

These studies add to the literature in humans on *NFX1*. They highlight that NFX1 contains motifs involved in gene and protein regulation and effects on drug therapies. They also complement the initial studies of NFX1 in immune pathway regulation and responses to inflammation.

## 4. NFX1 and NFXL1 Functions in Human Diseases

With an understanding of *NFX1* in normal physiology, it is important then to turn to studies of *NFX1* and its variants in pathophysiology and disease. First, in central nervous system development, a human variant of *NFX1* (*NFXL1*), which is a transcription factor with domain similarities to *NFX1*, was found to confer increased risk for a specific language impairment [22]. Higher expression of *NFXL1* was found in regions of the cerebellum that were associated with this diagnosis [23], and HLA class II genes regulated by *NFX1* were also found to correlate with these specific language impairments [24]. Second, in vascular studies, familial segregation analysis revealed that *NFX1* c.2519T>C (p.Leu840Pro) was associated with intracranial aneurysms, a cerebrovascular disorder; this *NFX1* point mutation was found in only cases and was absent among unaffected family members [25], linking *NFX1* as a genetic risk factor for these familial intracranial aneurysms. Third, in gastrointestinal development, an *NFX1* novel de novo variant c.1723G>A (p.Val575Met) was found to be one of the deleterious variants in human esophageal atresia, the most common malformation of the upper digestive tract [26], suggesting a functional significance to *NFX1* expression in upper digestive tract development and disease. Lastly, in metabolism and endocrinopathies, greater NFX1, with decreased HLA-DRA gene expression, was observed in obese adolescent individuals with insulin resistance compared to those who were insulin sensitive [27]. These studies highlight the normal function that *NFX1*, and *NFXL1*, may play in development, metabolism, and cellular or organ system physiology that can be disrupted by a modulation in expression or de novo novel variant gene expression. 

### 4.1. NFX1, High-Risk Human Papillomavirus, and Cancer Studies

All of the *NFX1* studies summarized above were conducted across species assuming normal biology, although exogenous environmental stressors were included. What is interesting to consider is how infectious agents could modulate the cell or organism, and how *NFX1* may participate in the dysregulation of cellular genes and pathways due to these infections. One infectious agent, human papillomavirus (HPV), has been researched in exquisite detail in epithelial cells, and many of the *NFX1* studies over the past 15 years have been performed in epithelial cells in the context of HPV. These seminal papers have led to a greater understanding of the splice variants of *NFX1* expressed in human epithelial cells, their conserved protein domains, their normal functions in cells, and the direct roles in supporting the viral life cycle of HPV and the oncogenic potential of high-risk (HR) HPV infections.

Human papillomaviruses infect epithelial cells, and those that are defined as high-risk are so based on their epidemiologic association with cervical cancer [28]. Of the more than a dozen HR HPV types causally linked to cancer, HPV type 16 causes half of all cervical cancers worldwide [28]. All cancers caused by HR HPVs universally express the viral oncogenes E6 and E7; however, neither of these genes have an enzymatic function. Therefore, they must partner with host cell proteins to drive oncogenesis. More than 15 years ago, a study to discover novel proteins that collaborate with, and bind directly to, the HR HPV type 16 E6 (16E6) viral oncogene identified *NFX1* [29]. Two isoforms of *NFX1* are expressed in epithelial cells, and they both were found to bind to the 16E6 oncoprotein [29,30].

### 4.2. Two Human NFX1 Isoforms in Epithelial Cells: NFX1-91 and NFX1-123

**Figure 1:**
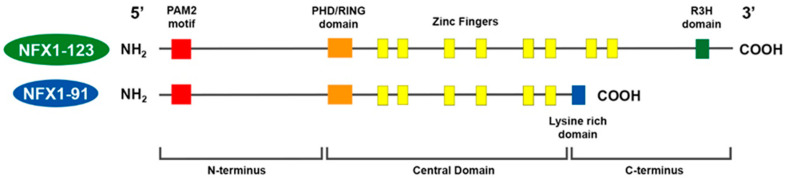
Model of *NFX1* splice variants in humans. NFX1-123 and NFX1-91 share a common N-terminus, with a PAM2 motif, and Central Domain, with a PHD/RING domain and six cysteine-rich zinc-like fingers. NFX1-91 has a truncated C-terminus that is lysine-rich. NFX1-123 has a unique C-terminus with two additional zinc-like fingers and an R3H domain.

The two splice variants of *NFX1* that are expressed in epithelial cells are named NFX1-91 and NFX1-123, based on their respective kilodalton masses [29]. NFX1-91 and NFX1-123 share a common N-terminus and central domain, but they have unique C-termini (Figure 1).

In the N-terminus, there is a PAM2 motif. This motif is necessary for proteins to bind to cytoplasmic poly(A) binding proteins. In the central domain, there is a PHD/RING domain that has an E3 ubiquitin ligase function. There are also six cysteine-rich zinc-like fingers, which are required for DNA binding and are conserved from the murine homolog. For NFX1-91, its C-terminus is short and is lysine-rich. For NFX1-123, its unique C-terminus includes two additional zinc-like fingers and an R3H domain, and this domain has putative single-stranded nucleic acid binding capabilities [31]. 

The shorter splice variant, NFX1-91, is rapidly targeted for ubiquitin-mediated degradation by 16E6 and the E3 ubiquitin ligase E6 Associated Protein (E6AP). 16E6 and E6AP polyubiquitinate the NFX1-91 protein at its lysine-rich unique C-terminus and target it for 26S proteasomal degradation; meanwhile, the longer isoform of *NFX1*, NFX1-123, is not [29]. These two isoforms of *NFX1* have distinct, and at times opposing, functions in gene regulation, driven by their protein domains and subcellular location. Those are described in detail below, as are how HR HPV disrupts and co-opts those functions of *NFX1* during oncogenesis. 

### 4.3. NFX1-91: A Transcriptional Regulator Destabilized by HR HPV

The NFX1-91 isoform is a nuclear protein that binds to an X1 box *cis*-element in the proximal *hTERT* promoter via its central domain. *hTERT* is the catalytic subunit of telomerase, and telomerase, as a key regulator of cellular immortalization, is universally activated in HPV-associated cancers [29]. NFX1-91 is a constitutive transcriptional repressor of *hTERT*, and it sits in complex with PKC-delta, a cellular senescence-inducing factor, and with the co-repressor complex mSin3A and histone deacetylase 1 at the *hTERT* promoter [32,33]. 16E6, and other E6 proteins from the beta HPV genus that can cause skin cancer, partner with E6AP to degrade NFX1-91, removing it from the *hTERT* promoter. This derepresses *hTERT* expression during an HPV infection [33] and extends the lifespan of epithelial cells in culture [34]. 

At the *hTERT* promoter, NFX1-91 functions as a transcriptional repressor, but NFX1-91 can also function as a transcriptional activator for other genes. Knockdown of NFX1-91 led to a reduction of NF-kB inhibitors such as p105, driving an induction of NF-kB-responsive genes [35]. The authors of this study offer a yet-to-be-tested hypothesis that different transcriptional co-regulators, partnering with NFX1-91, may drive this difference in activation versus repression of genes. It highlights the importance of the protein-partnerships between NFX1-91 and other transcriptional regulators. This holds true in protein partnerships and functions of the longer splice variant of *NFX1*, NFX1-123. 

### 4.4. NFX1-123: A Post-Transcriptional Regulator Stabilized by HR HPV

Since 16E6 and E6AP mediate the removal of NFX1-91, the more stable, longer splice variant of *NFX1*, NFX1-123, rises in importance during HR HPV infections. As NFX1-123 is not targeted by 16E6 and E6AP for rapid ubiquitin-mediated degradation like NFX1-91, and because NFX1-123 has opposing effects to NFX1-91 in HPV associated cancers, more recent studies have focused on defining and understanding the role NFX1-123 plays in cancers caused by HR HPV. A paper published in 2019 demonstrated that the deubiquitinase USP9X interacted with and stabilized the NFX1-123 protein through its efficient deubiquitination [36]. USP9X was increased in HPV-associated cancers, and specifically by 16E6 [37]; as such, preserving greater NFX1-123 expression, through augmented USP9X, may be an important function during cancer development and progression. These roles of NFX1-123, in the context of HR HPV and 16E6 specifically, are described in further detail below. 

In 2007, we were the first to identify that *NFX1* indeed had a conserved PAM2 motif in its N-terminus (Figure 1) [30]. We determined that the NFX1-123 protein-bound cytoplasmic poly(A) binding proteins (PABPCs) via this PAM2 motif, and together NFX1-123 and PABPCs synergistically augmented hTERT expression and telomerase activity in 16E6 expressing cells [30]. We then demonstrated that NFX1-123 colocalized with PABPCs in the cytoplasm but did not shuttle between nucleus and cytoplasm with them [38], so NFX1-123 played no direct role in transcriptional regulation of RNA translocation. Rather, we noted that NFX1-123 contained both a PAM2 motif and a unique R3H domain, and these protein domains were required to post-transcriptionally increase hTERT through binding and stabilization of its mRNA. The 5′ UTR of the hTERT mRNA was a required *cis*-element for NFX1-123 to maintain the post-transcriptional upregulation of hTERT [38] and to further increase hTERT and telomerase activity over time [39]. Therefore, NFX1-123 and its protein partnership with PABPCs led to increased hTERT expression and telomerase activity in HR HPV positive, or 16E6 positive, cells. This extension of growth and drive towards cellular immortalization by *NFX1*, during an HR HPV infection, echoes the extension of lifespan seen with the *stc* homolog in Drosophila.

### 4.5. NFX1-123: Increased in Epithelial Differentiation and Drives Differentiation Pathways

We discovered that NFX1-123 was not only normally expressed in epithelial cells but NFX1-123 itself increased during epithelial cell differentiation [40]. That increase is further augmented by 16E6, and together NFX1-123 and 16E6 upregulate downstream differentiation pathways and targets [40,41]. One example of this is Notch1.

Notch1, an important regulator of cell growth and differentiation, was found to be increased by NFX1-123 and 16E6; like hTERT, this increase depended on the PAM2 and R3H domains of NFX1-123 [42]. NFX1-123, with 16E6, increased expression of the Notch1 canonical pathway genes Hes1 and Hes5, and the increase in these genes by NFX1-123 required the presence and activation of the Notch1 receptor. Expression of the keratinocyte differentiation genes Keratin 1 and Keratin 10 were also increased by NFX1-123 and 16E6, but their upregulation was not directly linked to Notch1 receptor stimulation like Hes1 and Hes5 were. More intriguingly, the increase in keratinocyte differentiation induced by NFX1-123 with 16E6 was uncoupled from the growth arrest, increase in p21, and decrease in proliferative factor Ki67 typically seen during differentiation [41]. These findings led to the thesis that NFX1-123 normally regulates differentiation in epithelial cells and keratinocytes, and NFX1-123 itself is increased during differentiation as well [40]. However, the regulation of differentiation pathways can be co-opted by 16E6. Described in detail below, greater NFX1-123 has been shown to permit differentiation with continued growth and protection of longevity in the context of 16E6 co-expression—all of which are fundamental to the HPV viral life cycle and the oncogenesis of HPV associated cancers.

### 4.6. NFX1-123 Increased in Cervical Cancers and Co-Regulates Differentiation and Longevity

High expression of NFX1-123 has been demonstrated in HPV-positive cervical cancer cell lines [41] and in primary cervical cancers, which are nearly universally HPV-positive [39,43]. In normal keratinocytes expressing 16E6, greater expression of NFX1-123 was associated with extended longitudinal active cellular growth and augment hTERT expression along with telomerase activity [39]. These are all key steps (longevity and immortalization) to support the initiation and progression of HPV-associated cancers. 

In addition, HPV as a virus requires cellular differentiation to maintain a productive and long-term infection. NFX1-123 expression in keratinocytes mediated augmented activation of epithelial differentiation indirectly through Notch1 [41] and directly through the JNK signaling pathway [40] while still protecting their growth [41]. In cells with episomal HPV 16 genome, greater NFX1-123 correlated with greater expression of HPV 16 L1, the major capsid protein of HPV that is induced by the host cell’s differentiation [40]. These studies collectively demonstrated the link between cellular gene regulation (hTERT, Notch1, JNK/ERK) and cellular pathway regulation (growth, immortalization, differentiation). NFX1-123 and 16E6 support both the HPV lifecycle in a keratinocyte [40] and oncogenesis over time [39]. All of these genes and pathways are linked by the continued, and increasing expression, of NFX1-123.

### 4.7. NFX1-123: Downregulation of Inflammation and Immune Regulation with HR HPV

As a virus, HPV must avoid immunosurveillance and detection. Two studies have highlighted the collaborative role NFX1 plays in immune regulation by HR HPV. In one, whole-genome microarray analysis of keratinocytes stably expressing 16E6 and a tagged overexpressed form of NFX1-123 revealed the downregulation of pro-inflammatory cytokines and interferon-stimulated genes at mRNA and protein levels, indicating the requirement of NFX1-123 for immune regulation by HPV [44]. In a second, immunogenic epitopes of HR HPV type 45, analyzed by time core simulation, revealed an overlap between the antigenicity of HR HPV proteins and epitopes from several endogenous cellular proteins; NFX1 was included as one of many proteins and pathways that contained overlapping epitopes [45]. This dampens the immune response to HR HPV type 45 as the foreign epitopes could be seen as self—thus protecting the virus from detection and clearance. These studies also speak to the original work, now more than 25 years old, that identified *NFX1* as a gene involved in modulations of the immune response and supportive data from *NFX1* homolog studies.

## 5. NFX1 in HR HPV and Non-HR HPV Associated Cancers

*NFX1*, through the NFX1-91 and NFX1-123 splice variants, appears to play a critical role in driving HPV-associated cancers, with cervical cancer being the primary one studied to-date. However, six types of cancers are associated with HR HPV, including head and neck cancers. HPV-positive head and neck cancers are now the majority of head and neck squamous cell carcinomas (HNSCCs) diagnosed in the USA; since 2017, the Center for Disease Control and Prevention (CDC) has found rates of HPV-positive HNSCCs surpassing those of cervical cancers [46]. This makes identifying and understanding any common biology between cervical cancers and HPV-positive HNSCCs of increasing importance. In our recent studies, we confirmed NFX1-123 expression in HPV-positive HNSCCs was higher than that found in HPV-negative HNSCCs (manuscript in preparation). This indicated that, like in cervical cancer, NFX1-123 may have a specific significance in HPV-positive HNSCCs, and its high expression functions in a manner similar to that in cervical cancer [39,43].

*NFX1* also appears to be important in cancers that are not specifically caused by, or associated with, HR HPV infections. In clear cell renal cell carcinoma, downregulation of the *NFX1* was one of the positive regulators of hTERT expression [47]. In cases of esophageal squamous cell carcinoma, a missense variant of *NFX1* c.3350A>T (p.Asp1117Val) was found as well as loss of heterozygosity [48]. In breast cancer, MCF7 cells expressed high levels of NFX1 when compared to T47D cells [49]. Additionally, MCF7 cells were less responsive to Adriamycin treatment [50], suggesting that *NFX1* may be associated with Adriamycin resistance. Finally, in hepatocellular carcinoma, *NFX1* expression levels were noted as a biomarker that could separate complete response and partial response subsets of the patient after chemoembolization [51]. These studies all suggest that *NFX1* expression may be modulated in cancers, and this change is associated with key oncogenesis pathways and with differences in treatment responses.

## 6. Conclusions

*NFX1*, and specifically the NFX1-123 isoform, is expressed at high levels in cancers when compared to normal tissues. While this is clearly linked with two cancers that are HPV-associated (cervical and HNSCC), changes in *NFX1* expression can occur in other types of cancers. With that, it is interesting to consider that NFX1-123 may play a more universal role in oncogenesis, and HPV co-opts that functionality, specifically manipulating it during HPV-driven cancers. It is important to note as well that *NFX1*, and its homologs, disrupt drug interactions, affect growth, and protect against cellular and organismal stress—all functions often involved in cancer development and therapeutic resistance. As we described above, *FAP2* of *s. cerevisiae* competes with rapamycin to bind to FKBP12, the rapamycin receptor [8], and NFX1 in humans binds to Taxol [21]. In fruitflies, *stc* is required for embryonic development and extended lifespans of adults [3,4,5,6]. In plants, *NFX1* homologs protect against high temperature and drought stress [11] and blunt phytotoxin-induced defense response [13,14]. Further detailed mechanistic studies in drug resistance and stress tolerance are warranted to determine the specific functional significance of NFX1-123 in cancer development and cancer treatment outcomes.

**Figure 2:**
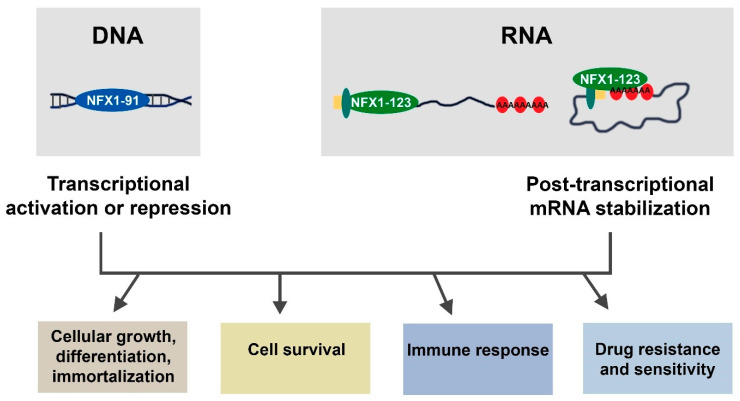
Regulation of gene expression by NFX1. Model of NFX1 (NFX1-91 and NFX1-123) mediated gene regulation. NFX1-91 binds to DNA to regulate gene expression either through transcriptional repression or activation. NFX1-123 binds to RNA to regulate gene expression through post-transcriptional regulation. NFX1-123 also interacts with other RNA binding and processing proteins, including cap-binding proteins (shown in yellow and teal) and cytoplasmic poly(A) binding proteins (shown in red). The cellular pathways affected by NFX1 are shown.

In humans, *NFX1* is a gene that produces nucleic acid-binding proteins (Figure 2).

The NFX1 proteins bind to DNA (NFX1-91) and RNA (NFX1-123), and they are involved in gene expression regulation through transcriptional repression, transcriptional activation, and post-transcriptional mRNA stabilization. These gene expression modulations by NFX1 lead to changes in cellular pathways and processes including cellular growth, differentiation, immortalization, cell survival, immune responses, and drug resistance and sensitivity. All of these—nucleic acid binding, gene expression changes, and cellular pathway regulation—are important to understand the role NFX1 plays in cellular and organismal biology, in disease, and cancer.
